# The Association between Externalizing and Internalizing Problems with Bullying Engagement in Adolescents: The Mediating Role of Social Skills

**DOI:** 10.3390/ijerph181910444

**Published:** 2021-10-04

**Authors:** Mariana Lopes de Sousa, Maria Manuela Peixoto, Sara Figueiredo Cruz

**Affiliations:** Centro de Investigação em Psicologia para o Desenvolvimento (Psychology for Positive Development Research Center), Lusíada University, 4100-348 Porto, Portugal; mmpeixoto@por.ulusiada.pt (M.M.P.); saracruz@por.ulusiada.pt (S.F.C.)

**Keywords:** adolescents, internalizing and externalizing problems, empathy, assertiveness, aggression, victimization

## Abstract

Bullying has severe public health consequences, due to its high prevalence worldwide and devastating effects on physical and mental health. Therefore, it is relevant to further understand the factors that contribute to the emergence and maintenance of bullying. This study aimed to examine the differential mediating role of social skills in the relationship between (i) externalizing problems and engagement in aggressive bullying behaviors, and (ii) internalizing problems and the engagement in victimization bullying behaviors. Participants were 669 Portuguese adolescents aged between 12 and 19 years. The Social Skills Improvement System-Rating Scales and the Scale of Interpersonal Behavior at School were used to assess social skills and the engagement in bullying behaviors, respectively. Boys scored higher on aggressive behaviors and externalizing problems. Girls reported higher scores on internalizing problems, communication, cooperation and empathy. Social skills differently mediated the association between behavior problems and engagement in bullying. While empathy negatively mediated the association between externalizing problems and aggressive bullying behaviors, assertiveness negatively mediated the relationship between internalizing problems and victimization bullying behaviors. The risk factors for engaging in bullying are discussed, and so are the protective ones, which may help to prevent bullying behaviors and reduce their negative impact.

## 1. Introduction

Adolescence is a critical developmental period, characterized by profound physical and psychological changes, as well as by the expansion and modification of interpersonal relationships [1,2,3]. Dealing with the complexity of the relationships established at school, as one of the most important contexts in which the development occurs, makes adolescents more prone to engage in conflicts with peers [4,5,6,7,8]. Although these conflicts can be expressed in different forms, bullying is considered the most common one [9].

Bullying is a significant public health concern, with devastating effects on physical and mental health [10,11,12] and strong educational, social and political implications. With a high incidence worldwide, one in three students (32%) reported being victims of bullying at school at least once in the last month [9]. Although physical bullying is decreasing in most countries, other forms of bullying (e.g., cyberbullying) are growing [9]. In Portugal, bullying is also frequent [13], with a mean of 39% of children and adolescents reporting having been bullied by their peers at least once in the last month [9]. In a study developed by the Health Behavior in School-aged Children [14], with a sample of 6997 Portuguese adolescents aged between 12 and 16 years, 6599 (94%) adolescents identified themselves as bullies, and 6598 (94%) as victims, which highlights the high incidence of this phenomenon and the relevance of understanding it more deeply.

Due to the widely supported effects of behavioral problems on engagement in bullying, more evidence is needed to understand how socioemotional adjustment problems impact violent interactions with peers. The association between internalizing and externalizing problems with bullying [15,16,17,18,19], as well as between social skills and bullying, are widely reported [17,20,21,22,23,24]. However, evidence on how socioemotional adjustment problems and social skills interact and predict engagement in bullying behaviors is lacking. To fill this gap, this study aimed to investigate the association between externalizing and internalizing problems with bullying engagement in adolescents by exploring the differential mediating role of social skills in this association. 

Bullying refers to aggressive behaviors that occur intentionally and repeatedly, over time, to harm others, in a context of observable and/or perceived power or inequity [19,25,26]. These systematic abusive behaviors aim to conquer, or maintain, a privileged social position [25]. 

Although direct aggression is the most obvious form of bullying behaviors, bullying also comes in indirect forms of aggression and victimization. The United Nations Educational, Scientific and Cultural Organization [9] proposed the distinction of four main types of bullying: (1) physical, (2) psychological, (3) sexual, and (4) cyberbullying. Physical bullying consists of persistent aggressions, such as making threats, kicking, hitting, hurting, pushing, shoving around or locking indoors, stealing, taking or destroying others’ personal belonging and coercing them to do things. Psychological bullying refers to verbal and emotional abusing, such as intentionally excluding or ignoring someone, insulting, teasing or spreading lies and rumors. Sexual bullying includes sexual jokes, comments or gestures to tease and humiliate someone. Cyberbullying consists of sending messages and treating others in a hurtful or cruel way through social media (i.e., texts, calls, video clips) or online (i.e., email, instant messaging, social networking, chatrooms). Despite its increasing prevalence, cyberbullying continues to be less frequent than the traditional forms of bullying [27]. 

Several risk and protective factors are associated with bullying behaviors [28,29]. Behavior problems and social skills are among these factors [17,28,29,30,31,32]. Internalizing and externalizing problems contribute to the maintenance and aggravation of bullying interactions [17,32,33,34]. Regarding social skills, specific abilities tend to have a differential effect on the engagement in bullying behaviors, depending on the adolescent’s role in these interactions (i.e., aggressor or victim) [31,35,36,37].

Social skills comprise interpersonal behaviors, leading to positive and adjusted responses, and avoiding negative reactions in social interactions, which allow being judged as socially competent by other/s [38,39,40]. According to Gresham et al. [40], six social skills can be defined: cooperation, assertiveness, responsibility, empathy, self-control and communication. Cooperation includes helping, sharing things and rule compliance. Assertiveness refers to behaviors that arise from one’s initiative (i.e., talking about oneself and requesting information from others) or from a response to others’ actions (i.e., responding to peer group pressure), as well as to expressions of respect for oneself and others. Responsibility is associated with the ability to communicate with adults and perform requested tasks, and with concern about oneself and others. Empathy comprises the interest and concern for feelings expressed by significant figures (i.e., parents, other significant adults, teachers) and the peer group. Self-control consists of adjusted regulation of emotions, in situations of conflict (i.e., showing an adaptive response to provocations and accepting corrective feedback from adults), as well as of compliance with rules and respect for imposed limits. Communication refers to the ability to initiate and/or maintain dialogues in a proper way, and use social rules and conventions (i.e., saying please and thank you).

Evidence supports the association between social skills and engaging in bullying [17,20,21]. The relationship between social skills and bullying has been mostly studied from the victim perspective [20,22,41,42]. However, when considering both aggression and victimization bullying behaviors, some social skills have a protective role when engaging in bullying, while others make adolescents more prone to engage in these abusive behaviors [31,35,36,37]. Bullies tend to exhibit higher levels of assertiveness and lower levels of empathy, cooperation and self-control [21,23,24,43]. Therefore, bullies, especially those who are indirect aggressors, do not lack all social skills, but only specific ones, as some of them can be very effective in manipulating situations for their advantage [44,45,46]. Contrary to bullies, bullying victims tend to show lower levels of assertiveness, but, as the aggressors, they are more likely to express difficulties in cooperation and self-control [21,47]. 

Internalizing problems refers to difficulties in controlling both emotions and cognitions, leading to anxiety/depression symptoms, being withdrawn, or somatic complaints [48,49]. Evidence shows that internalizing problems tends to predict the engagement in victimization behaviors [32,33,34,50]. Depressive and anxiety symptoms, self-harm, and suicidal ideation are common among victims of bullying [51,52,53,54,55,56,57].

Externalizing problems are also associated with difficulties in emotion and behavior regulation, but they tend to influence the context more negatively than internalizing problems [58,59]. Externalizing difficulties are usually related to the presence of aggressive, disruptive and acting-out behaviors [48,49]. Externalizing problems are closely associated with bullying engagement [17,50], and disruptive and antisocial behaviors, such as alcohol and substance abuse, which are commonly observed among bullies [15,33,60,61]. Adolescents with externalizing problems are more likely to experiencing difficulties in reading social cues and responding to complex interactions, which can explain their increased engagement in bullying [21,31,35,36,37,62]. 

The association between behavior problems, social skills and engagement in bullying behaviors is extensively supported [17,28,29,30,31,32]. Both behavior problems and social skills predict engagement in bullying [16,17,18,19,30,31]. However, to the best of our knowledge, the potential mediating role of social skills in the relationship between externalizing and internalizing problems with bullying has not yet been explored. To address this gap, this study examines the potential mediating role of social skills in the relationship between (i) externalizing problems and engaging in aggressive bullying behaviors; and (ii) internalizing problems and engaging in victimization bullying behaviors. It is expected that social skills differentially mediate the relationship between externalizing problems and aggressive bullying behaviors, and the relationship between internalizing problems and victimization bullying behaviors. Particularly, it is hypothesized that lower empathy and self-control and higher assertiveness mediate the relationship between externalizing and aggressive bullying behaviors, whereas lower assertiveness and cooperation mediate the relationship between internalizing and victimization bullying behaviors. 

## 2. Materials and Methods

### 2.1. Participants

Participants were 669 adolescents (385 girls and 284 boys) aged between 12 and 19 years (*M* = 14.58; *SD* = 1.44), attending schools within the Porto district, Portugal. Of these, 243 (36.3%) attended the 9th grade, 153 (22.9%) the 8th, 95 (14.2%) the 11th, 80 (12.0%) the 10th, 53 (7.9%) the 7th, and 41 (6.1%) the 12th. Boys were aged between 12 and 19 years (*M* = 14.64; *SD* = 1.43)—106 (37.3%) attended the 9th grade, 70 (24.6%) the 8th, 39 (13.7%) the 11th, 35 (12.3%) the 10th, 20 (7.0%) the 7th, and 14 (4.9%) the 12th. Girls were aged between 12 and 18 years (*M* = 14.53; *SD* = 1.45)—137 (35.6%) attended the 9th grade, 83 (21.6%) the 8th, 56 (14.5%) the 11th, 45 (11.7%) the 10th, 33 (8.6%) the 7th, and 27 (7.0%) the 12th.

Participants were eligible if they were Portuguese speakers without cognitive difficulties—identified by the teachers—so they could, autonomously, complete the questionnaires.

### 2.2. Measures

#### 2.2.1. Sociodemographic Questionnaire

Adolescents were invited to answer a sociodemographic questionnaire with questions about their age, sex, school grade and the city of residence. 

#### 2.2.2. Social Skills and Behavior Problems

The self-report version of Social Skills Improvement System-Rating Scales (SSIS-RS; [63,64]), was used to assess adolescents’ social skills and behavior problems. It consists of 75 items, assessed in a 4-point *Likert*-scale (from 0—never/almost never to 3—almost always/always). Two scales are obtained: social skills and behavior problems.

The social skills scale (composed of 46 items) consists of seven subscales: Communication (e.g., I am polite when I talk to other people), Cooperation (e.g., I follow the rules at school), Assertiveness (i.e., I ask questions when I have doubts), Responsibility (e.g., I am well-behaved), Empathy (e.g., I try to forgive when someone apologize.), Engagement (e.g., I get along with other children/adolescents.) and Self-Control (e.g., I try to find a good way to end a discussion). The behavior problems scale (composed by 29 items) consists of four subscales: Externalization (e.g., I hurt people when I feel angry), Bullying (e.g., I try to make others afraid of me), Hyperactivity/Attention Deficit (e.g., I am easily distracted) and Internalization (e.g., I get tired). The total scores for the Social Skills and Behavior Problems scales are obtained by summing the scores of the items of each subscale. 

Acceptable-to-good internal consistency results were observed for the social skills and behavior problems subscales: Communication (α = 0.78), Cooperation (α = 0.76), Assertiveness (α = 0.70), Responsibility (α = 0.80), Empathy (α = 0.83), Engagement (α = 0.81), Self-Control (α = 0.76), Internalization (α = 0.83) and Externalization (α = 0.81).

#### 2.2.3. Bullying Engagement

The Interpersonal Behavior at School (SIBS; [65]) scale was used to assess bullying engagement, both as an aggressor or as a victim. This questionnaire comprises 22 items describing aggression and victimization behaviors in bullying interactions. These items are assessed in a 4-point *Likert* scale (from 1 = never happens to 4 = it happens quite often). SIBS includes four scales: Verbal Aggression, Indirect Aggression, Verbal Victimization and Indirect Victimization. Verbal Aggression encompasses four items regarding verbal aggression while interacting with peers (e.g., I call names to my colleagues that hurt them). Indirect Aggression includes three items regarding aggressive behaviors aiming to withdraw and intimidate victims (e.g., I force other colleagues to do things they don’t want). Verbal Victimization includes four items referring to threats and verbal intimidation (e.g., My colleagues call me names that I don’t like). Indirect Victimization consists of three items concerning to being isolated from the group, threatened or intimidated by their peers (e.g., My colleagues ignore me). The score of each scale is obtained by summing the scores of the corresponding items. For the purpose of this study, the total score of Verbal Aggression and Indirect Aggression were summed to compute the total score of Aggression Bullying Behaviors and the scores of Verbal Victimization and Indirect Victimization were summed to obtain the total score of Victimization Bullying Behaviors. 

Good internal consistency results were observed for Aggression Bullying Behaviors (α = 0.83) and Victimization Bullying Behaviors (α = 0.85).

### 2.3. Procedure

This study was reviewed and approved by the University Ethics Committee from the authors affiliation. Schools in the Porto district, Portugal, were contacted, and invited to participate in the study. An information document describing the study goals, instruments and procedures, as well as ensuring the confidentiality and anonymity of the data collection, was sent by e-mail to the school directors that manifested interest in participating. After giving their consent, school directors selected a teacher, or a group of teachers, to mediate communication with adolescents.

Data collection occurred between October 2020 and June 2021, during the COVID-19 pandemic. Adolescents were asked to fill out the questionnaires online through the Google Forms platform, in the classroom under the supervision of the teacher/s. An e-mail was sent to the school directors with the link to complete the questionnaires, who forwarded it to the selected teacher/s. 

The teachers in the classroom provided adolescents with information regarding the study goals, instruments and required procedures, as well as ethical issues. Then, the informed consent was given to the adolescents so their parents, or alternative legal representatives or guardians, could authorize and consent to their participation in the study. Only adolescents who volunteered to be enrolled and whose parents consented to their participation received the link to the questionnaires and participated in this investigation. Data collection lasted approximately 15 to 20 min.

### 2.4. Data Analyses

Statistical analyses were conducted using IBM SPSS version 26.0. Descriptive statistics were computed for sample characterization, with calculation of means, standard-deviations, ranges, and frequencies. A Multivariate Analysis of Variance (MANOVA) with Bonferroni corrections was performed for assessing sex differences regarding all variables. Pearson’s correlation coefficients were calculated to examine the association between all variables (behavior problems, social skills and bullying behaviors). After checking statistical assumptions and correlation coefficients between all variables, two mediation models were conducted, using model 4 from PROCESS macro 3.5.2 for IBM SPSS [66], with Bootstrapping Confidence Intervals. These models assessed the relationships between (1) internalizing problems and aggressive bullying behaviors and (2) externalizing problems and victimization bullying behaviors, both mediated by social skills (communication, cooperation, assertiveness, responsibility, empathy, engagement, and self-control). Indirect effects were tested with 5000 bootstrap samples based on the 95% Bias-Corrected Bootstrap Confidence Intervals (95% BCBCI; [67]). According to exploratory analysis, variable sex should be controlled and was used as a covariate in the mediation models. Effect-size interpretation criteria (small—0.01; medium—0.09; large—0.25) was performed according to Preacher and Kelley [68], and percentage of total effect mediated was also assessed [69].

## 3. Results

### 3.1. Social Skills, Behavior Problems and Bullying

Means, standard deviations, and ranges of the responses regarding social skills, internalizing and externalizing problems, and aggressive and victimization bullying behaviors are illustrated at Table 1. Multivariate analysis of variance revealed significant main effects for sex, Wilks’ lambda = 0.82, *F*(11,657) = 12.83, *p* < 0.001, partial *η*^2^ = 0.177. As presented at Table 2, the univariate analysis indicated that boys scored significantly higher on aggressive behaviors (*p* < 0.001) and externalizing problems (*p* = 0.011) compared to girls, whereas girls reported higher scores on internalizing problems (*p* < 0.001), communication (*p* < 0.001), cooperation (*p* < 0.001), responsibility (*p* < 0.001), and empathy (*p* < 0.001), when compared with boys. Pearson’s correlation coefficients for all variables are presented at Table 2.

### 3.2. Mediation Role of Social Skills in the Relation between Externalizing Problems and Aggressive Bullying Behaviors

The mediation model is grounded on a variable (externalizing problems) which is theoretically suggested to predict and influence an outcome (aggressive bullying behaviors) through a set of mediating variables (social skills). Two pathways were defined by which externalizing problems may predict aggressive bullying behaviors, controlling for sex (i.e., covariable). The mediators entered into the model were communication skills, cooperation, assertiveness, responsibility, empathy, and self-control (engagement was excluded due to nonsignificant statistical correlation with externalizing problems and aggressive bullying behaviors). The mediation model explained 28.8% of the variance of aggressive bullying behaviors, which was significant, R^2^ = 0.288, *F*(8,660) = 33.40, *p* < 0.0001.

**Figure 1:**
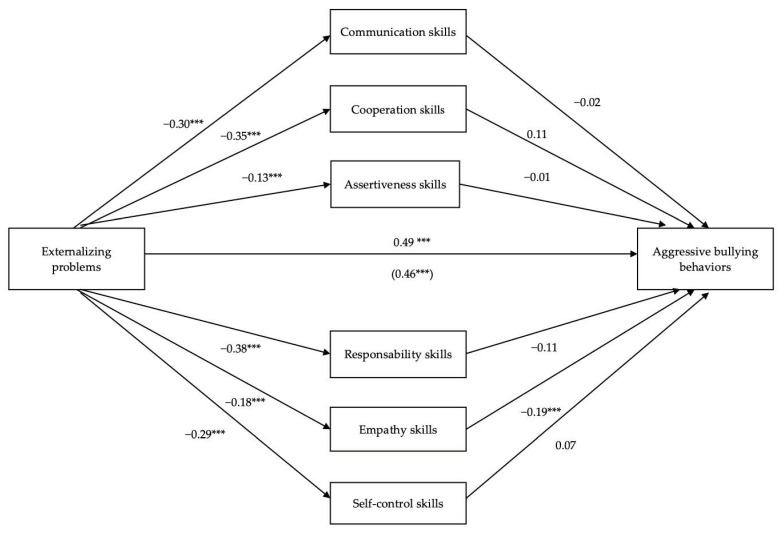
The mediating role of social skills in the relation between externalizing problems and aggressive bullying behaviors (N = 669). *** *p* < 0.001.

The regression of externalizing problems on aggressive bullying behaviors was statistically significant, *β* = 0.49, *SE* = 0.01, *t* = 14.45, *p* < 0.0001; 95% BCBCI 0.18–0.24, and the covariable sex revealed a statistically significant effect, *β* = 0.10, *SE* = 0.17, *t* = 2.90, *p* = 0.004; 95% BCBCI 0.16–0.81, with boys reporting more externalizing problems and being more likely to engaging in aggressive bullying behaviors. The regression of externalizing problems on mediators was statistically significant (communication, *β* = −0.30, *SE* = 0.02, *t* = −8.40, *p* < 0.0001; 95% BCBCI −0.20–−0.12; cooperation, *β* = −0.35, *SE* = 0.02, *t* = −9.93, *p* < 0.0001; 95% BCBCI −0.26–−0.18; assertiveness, *β* = −0.13, *SE* = 0.03, *t* = −3.28, *p* = 0.001; 95% BCBCI −0.14–−0.04; responsibility, *β* = −0.38, *SE* = 0.02, *t* = −10.93, *p* < 0.0001; 95% BCBCI −0.28–−0.20; empathy, *β* = −0.18, *SE* = 0.02, *t* = −4.74, *p* < 0.0001; 95% BCBCI −0.15–−0.06; and self-control, *β* = −0.29, *SE* = 0.02, *t* = −7.65, *p* < 0.0001; 95% BCBCI −0.24–−0.14). The regressions of mediators on aggressive bullying behaviors were statistically significant for empathy, *β* = −0.19, *SE* = 0.04, *t* = −3.81, *p* = 0.0001; 95% BCBCI −0.20–−0.07, revealing that adolescents with more externalizing problems, who express less empathy skills, engage in more aggressive bullying behaviors. Finally, the regression of externalizing problems on aggressive bullying behaviors after controlling for social skills (mediators) was significant, β = 0.47; *SE* = 0.02, *t* = 12.71, *p* < 0.0001; 95% BCBCI 0.17–0.23 (Figure 1). The mediation effect size was 0.01. Regarding percentage of mediation, 7.28% of the total effect of externalizing problems on aggressive bullying behaviors was mediated by empathy. 

### 3.3. Mediating Role of Social Skills in the Relationship between Internalizing Problems and Victimization Bullying Behaviors

Likewise, the mediation model is supported on a variable (internalizing problems) which is theoretically suggested to predict and influence an outcome (victimization bullying behaviors) through a set of mediating variables (social skills). Two pathways are defined by which internalizing problems may predict victimization bullying behaviors, controlling for sex (i.e., covariable). The mediators entered into the model were assertiveness, empathy, engagement, and self-control (communication, cooperation, and responsibility were excluded, due to the non-significant statistical correlation with internalizing problems). The mediation model explained 26.3% of the variance of aggressive bullying behaviors, which was significant, R^2^ = 0.263, *F*(6,662) = 39.40, *p* < 0.0001. 

**Figure 2:**
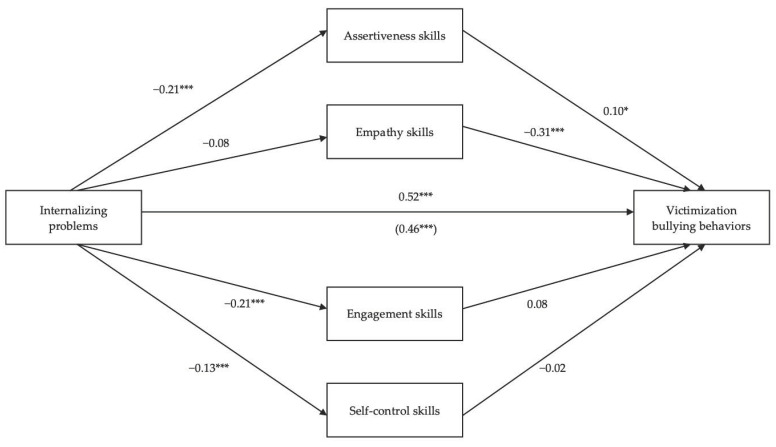
The mediating role of social skills in the relationship between internalizing problems and victimization bullying behaviors (N = 669). * *p* <0.05; *** *p* < 0.001.

The regression of internalizing problems on victimization bullying behaviors was statistically significant, *β* = 0.52, *SE* = 0.02, *t* = 13.05, *p* < 0.0001; 95% BCBCI 0.21–0.29, and the covariable sex revealed a statistically significant effect, *β* = 0.10, *SE* = 0.23, *t* = 2.90, *p* = 0.004; 95% BCBCI 0.22–1.14, with girls reporting more internalizing problems and being more likely to engage in victimization bullying behaviors The regression of internalizing problems on mediators was statistically significant (assertiveness, *β* = −0.21, *SE* = 0.03, *t* = −5.29, *p* < 0.0001; 95% BCBCI −0.19–−0.09; engagement, *β* = −0.21, *SE* = 0.03, *t* = −5.32, *p* < 0.0001; 95% BCBCI −0.20–−0.09; and self-control, *β* = −0.13, *SE* = 0.02, *t* = −3.27, *p* = 0.001; 95% BCBCI −0.13–−0.03). The regressions of mediators on victimization bullying behaviors were statistically significant for assertiveness, *β* = 0.10, *SE* = 0.03, *t* = 2.37, *p* = 0.02; 95% BCBCI 0.01–0.15, and for empathy, *β* = −0.31, *SE* = 0.04, *t* = −6.66, *p* < 0.0001; 95% BCBCI −0.38–−0.21. Finally, the regression of internalizing problems on victimization bullying behaviors after controlling for social skills (mediators) was significant, β = 0.46; *SE* = 0.02, *t* = 13.05, *p* < 0.0001; 95% BCBCI 0.21–0.29 (Figure 2). The mediation effect size was 0.01. Regarding percentage of mediation, 4.57% of the total effect of internalizing problems on victimization bullying behaviors was mediated by assertiveness, revealing that adolescents with more internalizing problems, who express more assertiveness skills, engage more often in victimization bullying behaviors. 

## 4. Discussion

This study examined the potential mediating role of social skills in the relationship between (i) externalizing problems and engaging in aggressive bullying behaviors, and between (ii) internalizing problems and engaging in victimization bullying behaviors, in a sample of Portuguese adolescents. As expected, a differential mediating role of social skills was observed in the relationship between behavioral problems and the engagement in aggressive or victimization bullying behaviors. While empathy mediated the relationship between externalizing problems and engagement in aggressive bullying behaviors, assertiveness mediated the relationship between internalizing problems and engagement in victimization bullying behaviors.

### 4.1. Sex Differences Regarding Social Skills, Behavior Problems and Bullying

Results revealed differences between boys and girls regarding social skills, behavioral problems, and bullying. Boys reported more externalizing problems and more involvement in aggressive behaviors than girls. On the other hand, girls reported more internalizing problems, as well as more social skills—communication, cooperation, responsibility and empathy—than boys.

These results are in line with the existing literature, showing that externalizing problems are more common in boys, while internalizing problems are more frequent in girls [70,71,72,73]. These results also support the evidence that girls tend to read social cues more accurately and to respond in an appropriate way to social interactions [17,74,75], as they are more likely to exhibit higher levels of cooperation [76] and empathy skills, compared to boys [77].

### 4.2. Association between Externalizing Problems and Aggressive Bullying Behaviors Mediated by Social Skills

These results indicated a positive association between externalizing problems and engagement in aggressive bullying behavior. The effect of externalizing problems on engagement in aggressive behaviors is extensively described [17,33,61,62]. In addition, boys presented greater externalizing problems, which were negatively associated with social skills, particularly poorer communication, cooperation, assertiveness, responsibility, empathy and self-control abilities. This is in line with other evidence indicating that socioemotional maladjustment may be related to dysfunctions in social abilities, promoting involvement in bullying [44,45,46].

Importantly, the association between externalizing problems and aggressive bullying behaviors was mediated by empathy. In accordance with other evidence, boys who present lower empathy levels tend to engage in more aggressive behaviors [78]. This is further corroborated by the negative association observed between empathy and aggressive bullying behaviors. Evidence is consistent in demonstrating that those who cannot be interested or demonstrate concern about others’ feelings are more likely to enroll in hostile behaviors [43].

This result may yield important cues for interventional approaches. It suggests that empathy may be used as a protective factor for adolescents who exhibit externalizing problems. It may be that increasing interest and concern about others, thus express more empathy within peer interactions, may prevent engagement in aggressive bullying behaviors. Hence, empathy may act as buffer [79], as several studies support the positive association of empathic distress, or experiencing concern for others, with prosocial behavior (e.g. [80,81,82]). Adolescents who display increased empathy are more effective in reading and responding to others’ emotions, possibly leading them to deal with conflicts with their peers more effectively by employing well-adjusted social coping mechanisms and emotionally regulated responses [83]. 

### 4.3. Association between Internalizing Problems and Victimization Bullying Behaviors Mediated by Social Skills

A positive association between internalizing problems and victimization bullying behaviors was observed. Girls reported more internalizing problems, which were associated with poorer assertiveness, engagement, and self-control abilities. This is in accordance with studies indicating that poorer social skills may promote depressed/anxious states, which, in turn, promotes their role as victims of bullying [84]. 

Interestingly, results show that the relationship between internalizing problems and victimization bullying behaviors was positively mediated by assertiveness, contrarily to expected. It may be that internalizing symptoms, such as anxiety and depression, are possibly associated with a cognitive bias in processing information in social interactions [85,86]. This may lead adolescents to use social skills, and specifically assertiveness, in non-adaptative ways and increase the probability of being involved in conflicts with their peers, making them more prone to be victims of bullying. In line with this, the internalizing–externalizing problems’ comorbidity should also be considered, as adolescents who exhibit more socioemotional maladjustment are more likely to be less accurate while reading social interactions and, consequently, in using social skills [4,87]. Hence, they are potentially more vulnerable to be enrolled in conflicts with their peers, namely being victimized in bullying interactions. 

### 4.4. Limitations and Future Studies

This study has some limitations. The first refers to the potential geographical bias, as only adolescents from the Porto district participated in this study. In future studies, geographical representativeness should be ensured to replicate these findings. In addition, data were collected online, preventing identification and resolution of potential difficulties during the completion of the questionnaires. Hence, it would be important to administer the questionnaires face to face, to monitor the adolescents and clarify their doubts more closely. Furthermore, only the adolescents’ perception on their bullying behaviors, social skills and behavior problems were considered. Therefore, future research should broaden the range of informants, including parents and teachers, to achieve a more accurate, valid, and ecological approach.

## 5. Conclusions

Considering bullying as a major public health concern [10,11,12] which demands specialized interventions for preventing its incidence, current findings are of utmost relevance and have practical implications for psychologists working with adolescents in different contexts (e.g., clinical, school, community, and social), as it suggests specific social skills that should be enhanced and promoted in individual and group interventions with adolescents. For adolescents with externalizing problems and who engage in aggressive bullying behaviors, interventions should focus on developing empathy skills to foster a compassionate understanding of emotions, feelings, thoughts, and behaviors of others, encouraging interactions supported by kindness, solidarity and engagement, whereas for adolescents with internalizing problems and who are often victimized in bullying situations, interventional programs should emphasize assertiveness skills, such as the expression of one’s emotions, feelings, beliefs and desires in a self-confident and effective way. Assertiveness is a marker of self-efficacy and a key component of self-advocacy, when interacting with peers. Promoting more realistic self-image, less self-blame and strategies to speak up against victimization may contribute to reducing engagement in bullying behaviors [84]. 

This study extends the existing evidence by exploring the differential impact of social skills on the relationship between behavioral problems and bullying behaviors. These results highlight the importance of developing promotional, preventive, and remediation interventions in schools to mitigate socioemotional adjustment problems as psychopathology precursors, and to foster social skills, to prevent bullying behaviors and reduce their effects on peer interactions, as well as on adolescents’ mental and physical health. These interventions will possibly help children and adolescents in dealing with behavioral problems, as well as to communicate and solve conflicts with their peers more effectively, thus contributing to their socioemotional adjustment. Reducing bullying behaviors is imperative, and current findings suggest different pathways to minimize both aggression and victimization bullying behaviors through endorsing and promoting social skills in adolescents.

In addition, the present study emphasizes the need to conduct further studies to deeper understand how specific internalizing and externalizing symptoms, such as anxiety, depression, oppositional and defiant behaviors, interact with certain social skills in predicting engagement in bullying behaviors.

## Figures and Tables

**Table 1:** Means, standard deviations, and range values, as well as between-sex differences results for internalizing and externalizing problems, aggressive and victimization bullying behaviors, and social skills.

Variables	Total (*N* = 669)		Boys (*n* = 284)		Girls (*n* = 385)		Between-Sex Differences *		
	M (SD)	Range	M (SD)	Range	M (SD)	Range	F(1667)	*p*	*η* ^2^
Internalizing problems	11.55 (6.06)	0.00–30.00	9.90 (5.67)	0.00–26.00	12.77 (6.07)	0.00–30.00	38.72	<0.001	0.055
Externalizing problems	8.37 (565)	0.00–36.00	9.01 (5.95)	0.00–36.00	7.89 (5.39)	0.00–36.00	6.46	0.011	0.010
Aggressive bullying behaviors	8.32 (2.47)	7.00–28.00	8.74 (2.74)	7.00–28.00	8.01 (2.20)	7.00–27.00	14.31	<0.001	0.021
Victimization bullying behaviors	9.38 (3.26)	7.00–28.00	9.36 (3.36)	7.00–28.00	9.39 (3.19)	7.00–28.00	0.02	0.886	<0.001
Communication skills	14.73 (3.03)	0.00–18.00	13.98 (3.40)	0.00–18.00	15.28 (2.59)	0.00–18.00	31.33	<0.001	0.045
Cooperation	15.67 (3.55)	0.00–21.00	14.75 (3.94)	0.00–21.00	16.34 (3.07)	0.00–21.00	34.51	<0.001	0.049
Assertiveness	11.67 (3.99)	0.00–21.00	11.46 (4.19)	0.00–21.00	11.83 (3.84)	0.00–21.00	1.39	0.240	0.002
Responsibility	16.81 (3.53)	0.00–21.00	15.86 (4.01)	0.00–21.00	17.51 (2.94)	0.00–21.00	37.66	<0.001	0.053
Empathy	14.29 (3.41)	0.00–18.00	13.14 (3.74)	0.00–18.00	15.13 (2.87)	0.00–18.00	60.93	<0.001	0.084
Engagement	14.36 (4.31)	0.00–21.00	14.07 (4.49)	0.00–21.00	14.58 (4.17)	0.00–21.00	2.36	0.125	0.004
Self-control	10.73 (3.71)	0.00–18.00	10.82 (3.98)	0.00–18.00	10.66 (3.51)	0.00–18.00	0.32	0.574	<0.001

Note. Values are reported for the total sample (*N* = 669), and for boys (*n* = 285) and for girls (*n* = 385), separately. * Data for univariate tests (Multivariate analysis, *F*(11,657) = 12.83, *p* < 0.001, partial *η*^2^ = 0.177).

**Table 2:** Pearson’s correlation coefficients between internalizing and externalizing problems, aggressive and victimization bullying behaviors, and social skills (N = 669).

Variables	1.	2.	3.	4.	5.	6.	7.	8.	9.	10.	11.
1. Internalizing problems	-										
2. Externalizing problems	0.37 ***	-									
3. Aggressive bullying behaviors	0.19 ***	0.50 ***	-								
4. Victimization bullying behaviors	0.44 ***	0.39 ***	0.56 ***	-							
5. Communication skills	−0.02	−0.32 ***	−0.27 ***	−0.17 ***	-						
6. Cooperation	−0.01	−0.37 ***	−0.25 ***	−0.13 ***	0.73 ***	-					
7. Assertiveness	−0.018 ***	−0.13 **	−0.13 **	−0.11 **	0.51 ***	0.51 ***	-				
8. Responsibility	−0.01	−0.40 ***	−0.31 ***	−0.17 ***	0.77 ***	0.80 ***	0.49 ***	-			
9. Empathy	0.14 ***	−0.20 ***	−0.27 ***	−0.17 ***	0.67 ***	0.61 ***	0.48 ***	0.65 ***	-		
10. Engagement	−0.18 ***	−0.02	−0.05	−0.12 **	0.49 ***	0.40 ***	0.53 ***	0.39 ***	0.48 ***	-	
11. Self-control	−0.13 **	−0.28 ***	−0.15 ***	−0.15 ***	0.48 ***	0.49 ***	0.37 ***	0.52 ***	0.44 ***	0.31 ***	-

Note. ** *p* < 0.01; *** *p* < 0.001.

## Data Availability

The data that support the findings of this study is available upon request to the corresponding author. The data is not publicly available due to privacy or ethical restrictions.
